# Classification of neurodegenerative diseases using brain effective connectivity and machine learning techniques: a systematic review

**DOI:** 10.3389/fneur.2025.1581105

**Published:** 2025-05-22

**Authors:** Ying-Fang Wang, Yuan Huang, Xiao-Yi Chang, Si-Ying Guo, Yu-Qi Chen, Ming-Zhu Wang, Kai-Le Liu, Fang-Fang Huang

**Affiliations:** Department of Preventive Medicine, College of Basic Medicine and Forensic Medicine, Henan University of Science and Technology, Luoyang, China

**Keywords:** Alzheimer’s disease, brain effective connectivity, classification model, deep learning, electroencephalogram, functional magnetic resonance imaging, machine learning, neurodegenerative diseases

## Abstract

**Background:**

Effective connectivity (EC) refers to the directional influences or causal relationships between brain regions. In the field of artificial intelligence, machine learning has demonstrated remarkable proficiency in image recognition and the complex dataset analysis. In recent years, machine learning models leveraging EC have been increasingly used to classify neurodegenerative diseases and differentiate them from healthy controls. This review aims to comprehensively examine research employing EC—derived from techniques such as functional magnetic resonance imaging, electroencephalography, and magnetoencephalography—in conjunction with machine learning methods to classify neurodegenerative diseases.

**Methods:**

We conducted a literature search in accordance with the Preferred Reporting Items for Systematic Reviews and Meta-Analyses (PRISMA) guidelines, collecting articles published prior to June 13, 2024, from the PubMed and Embase databases.

**Results:**

We selected 16 relevant studies based on predefined inclusion criteria: six focused on Alzheimer’s disease (AD), six on mild cognitive impairment (MCI), one on Parkinson’s disease (PD), two on both AD and MCI, and one on both AD and PD. We summarized the methods for EC feature extraction and selection, the application of classifiers, validation techniques, and the accuracy of the classification models.

**Conclusion:**

The integration of EC with machine learning techniques has demonstrated promising potential in the classification of neurodegenerative diseases. Studies have shown that combining EC with multimodal features such as functional connectivity offers novel approaches to enhancing the performance of classification models.

## 1 Introduction

Neurodegenerative diseases are characterized by cognitive decline, severe motor disability, and dementia. These diseases include Parkinson’s disease (PD), Alzheimer’s disease (AD), Huntington’s disease, and amyotrophic lateral sclerosis (1). Furthermore, mild cognitive impairment (MCI), an antecedent to AD, is categorized into early MCI (EMCI) and late MCI (LMCI) (2). Recently, MCI has attracted considerable attention in clinical practice and research, owing to its progression to AD at an annual rate of approximately 10–15% (3). The incidence of neurodegenerative diseases escalates with age, significantly impairing the quality of life and survival rates of the elderly (4). The causes of neurodegenerative diseases remain unclear, and current treatment options are limited. However, timely classification and diagnosis, followed by appropriate treatment, can significantly improve patients’ quality of life (5–8). Moreover, the current lack of methods for classifying and diagnosing neurodegenerative diseases complicates the identification and timely intervention of these conditions (9). Current approaches predominantly depend on clinical symptoms (10). For instance, in AD, the manifestation of dementia symptoms typically leads to confirmation through neuroimaging techniques and cerebrospinal fluid assessments, revealing neuronal loss, and abnormal accumulation of amyloid-β and tau proteins, and temporal lobe cortical atrophy (11, 12). Similarly, the diagnosis of PD primarily involves an evaluation of the patient’s symptoms, medical history, physical examination, and response to dopamine therapy to differentiate it from other conditions and healthy individuals (13). Research indicates that identifying appropriate neural biomarkers is crucial for accurate disease identification (14). Employing neuroimaging techniques and electrophysiological biomarkers for disease classification can enhance treatment outcomes and slow disease progression (15).

Brain connectivity derived from imaging technologies elucidates the internal mechanisms of brain function, thereby expanding research avenues for the classification of neurodegenerative diseases (16, 17). Brain connectivity can be categorized into functional connectivity (FC) and effective connectivity (EC), both of which can be extracted through the analysis of functional magnetic resonance imaging (fMRI), electroencephalography (EEG), and magnetoencephalography (MEG) data. FC serves as a widely recognized physiological biomarker for diagnosing neurodegenerative diseases, illustrating the synchronous activity or correlation among different brain regions during resting or task states (18). It reflects the brain’s functional organization and network architecture by assessing inter-regional correlations, thereby unveiling connectivity alterations in specific brain regions associated with these diseases and facilitating the differentiation between healthy controls (HC) and those affected (19, 20). However, FC cannot provide information on directional interactions within brain networks, as correlations do not indicate any causal relationships or directionality between sites (21, 22). In contrast, EC reveals the causal effects and topological relationships of neural activities between different brain regions, offering valuable insights into the functional organization of the brain (23). Unlike FC, EC can identify lagged relationships between different brain areas, thus better elucidating the interaction mechanisms within the brain’s internal networks (24). Many neurodegenerative diseases may affect specific information transmission pathways in the brain at an early stage (25, 26). By analyzing changes in EC, it is possible to differentiate these diseases and develop classification models.

Machine learning is a technology that uses algorithms to learn from data and extract patterns to make predictions or decisions, while deep learning, a subset of machine learning, employs multilayer neural networks to model complex data and perform feature extraction (27, 28). Developing classification models based on brain EC using machine learning and deep learning techniques has become a cutting-edge approach for identifying and diagnosing neurodegenerative diseases. Extracted brain EC features can be used to train machine learning or deep learning models for the classification and diagnosis of neurodegenerative diseases. The models’ generalization capability and clinical application value are evaluated through cross-validation and validation with multicenter data. For example, Zhao et al. (29) developed a classification model using machine learning with EC features to distinguish AD from HC, while Qiao et al. (30) employed deep learning with EC features for the same purpose. However, a comprehensive review of these studies is currently lacking.

Therefore, the primary objective of this review is to systematically examine and summarize recent advances in constructing neurodegenerative disease classification and diagnosis models based on brain EC. This review provides a comprehensive overview of the current research landscape, with a particular focus on the various methodologies and techniques employed in these models, such as the extraction and identification of brain EC features and the application of machine learning and deep learning techniques. Many studies have compared different EC estimation methods or proposed new approaches, while researchers have also explored various machine learning techniques to enhance classification accuracy. Consequently, this review emphasizes the estimation methods for EC and the selection of machine learning approaches, aiming to promote the clinical application of classification and diagnosis models based on brain EC and machine learning methods, ultimately offering new tools and strategies for the classification of neurodegenerative diseases.

## 2 Materials

### 2.1 Information sources

**Figure 1:**
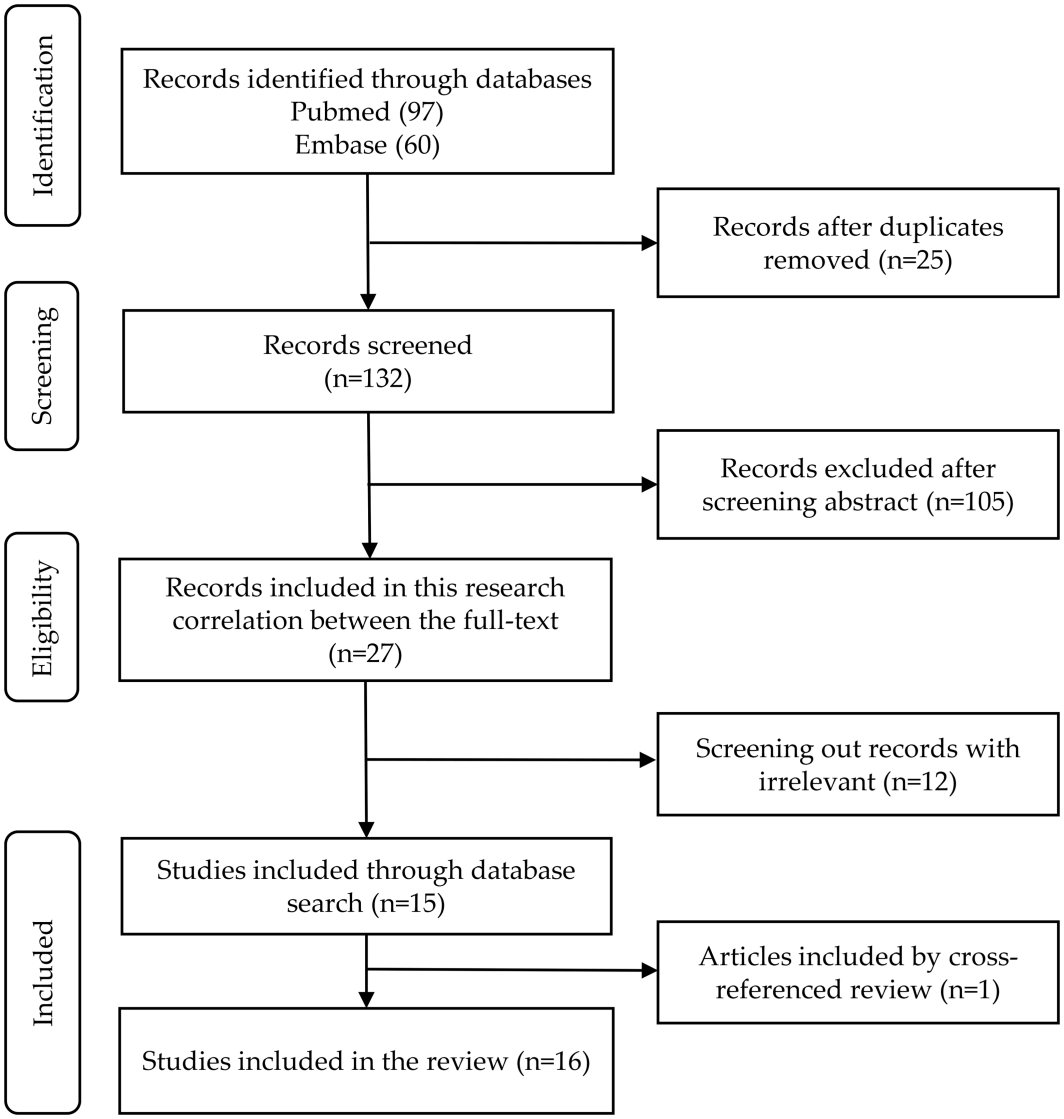
PRISMA flow diagram of literature search.

This review retrieved and screened literatures according to the Preferred Reporting Items for Systematic Reviews and Meta-Analyses (PRISMA) guidelines (31). PRISMA provides a standardized process for systematic reviews and meta-analyses, ensuring transparency and reproducibility in literature screening. This review gathered potentially relevant studies from two databases, including PubMed and Embase. After deleting the duplicate articles, two of the co-authors (YFW and YH) independently screened the articles based on the titles and abstracts for potential inclusion into this review. After reading the full text, articles agreed upon by both the authors were considered for the manuscript synthesis. In the event of a disagreement, a third researcher (FFH) will be invited to join the discussion and make the final determination. The steps of the screening, including literature exclusion reasons, were meticulously documented using a flow diagram (Figure 1).

### 2.2 Search strategy

The literature retrieval terms were (“neurodegenerative disease” OR “Alzheimer’s disease” OR AD OR “Parkinson’s disease” OR PD OR “mild cognitive impairment” OR MCI OR “Huntington’s disease” OR HD OR “Amyotrophic Lateral Sclerosis” OR ALS) AND (“effective connectivity” OR “effective brain connectivity” OR “Granger causality” OR “autoregressive model” OR “partial directed coherence” OR PDC OR “direct transfer function” OR DTF OR “transfer entropy” OR TE OR “dynamic causal” OR DCM OR “structural equation” OR SEM OR “Ornstein-Uhlenbeck” OR OUM OR “Bayesian network” OR BNM) AND (“functional magnetic resonance imaging” OR fMRI OR electroencephalogram OR EEG OR magnetoencephalography OR MEG) AND (Classif* OR predict* OR diagnos* OR identif* OR distinguish* OR “machine learning” OR “deep learning” OR “multivariate pattern analysis” OR “support vector machine” OR SVM OR “convolutional neural network” OR CNN OR “graph neural network” OR GNN OR “graph convolutional network” OR GCN) AND (accuracy). Articles published up to June 13, 2024 were collected.

### 2.3 Inclusion and exclusion criteria

This review encompasses original, peer-reviewed articles that adhere to the following criteria: (1) The article must be written in English; (2) The study must be based on EC features and employ machine learning or deep learning techniques to construct classification models, enabling the differentiation between patients and HC as well as the distinction among various neurodegenerative diseases. Exclusion criteria include: studies that extract EC without performing classification; studies that do not utilize machine learning or deep learning for model construction; as well as reviews and case reports.

### 2.4 Data extraction

Two authors (YFW and YH) extracted data from the included studies using a standardized data extraction form. The extracted data included the first author, publication year, data type (fMRI, EEG, or MEG), study features, EC estimation methods, classifiers, validation methods, classification groups, and model performance (accuracy). Any disagreements were resolved through discussion or by consulting another author (FFH).

## 3 Results

One hundred and fifty-seven articles were retrieved from PubMed and Embase, and 25 duplicates were discarded. After screening titles and abstracts, 105 irrelevant articles were excluded. Following a full-text review, 12 additional articles were excluded, leaving 15 articles that met the inclusion criteria—Six papers focus on constructing classification models to distinguish AD from HC, five on differentiating MCI from HC, one on distinguishing PD from HC, two on distinguishing between AD, MCI, and HC, and one on differentiating AD, PD, and HC. Additionally, one more article on MCI was included by cross-referenced review, bringing the total to 16 articles.

## 4 Extraction of EC features

### 4.1 Data preprocessing

The EC features were estimated using fMRI, EEG, or MEG data. It is essential to apply modality-specific preprocessing techniques. These methods must account for the physiological characteristics and noise patterns of different data types to extract high-quality time series signals.

#### 4.1.1 Preprocessing of fMRI data

Twelve studies (29, 30, 32–41) included in this review employed fMRI data to estimate EC and exhibited a highly consistent preprocessing workflow encompassing the following key steps. First, the initial time points were discarded to mitigate magnetization effects. This was followed by slice timing and motion correction. For spatial normalization, data were registered to the Montreal Neurological Institute standard space. To enhance the signal-to-noise ratio, the majority of studies implemented spatial smoothing with a Gaussian kernel. Subsequently, linear regression was performed to remove confounding factors, including cerebrospinal fluid, white matter signals, and head motion parameters, while detrending was applied to suppress low-frequency drift. Finally, a band-pass filter was implemented to preserve the target frequency range. These analyses were primarily conducted using the Statistical Parametric Mapping (SPM) (42) and Data Processing Assistant for Resting-State fMRI (DPARSF) (43) toolboxes, with slight variations across studies in parameters such as smoothing kernel size and covariate selection.

#### 4.1.2 Preprocessing of EEG data

Three studies (44–46) incorporated in this analysis employed EEG data, with preprocessing primarily focused on noise reduction and physiological artifact elimination. The protocol initiated with data import and format conversion, followed by verification of electrode positioning and channel labeling. Subsequent signal processing involved downsampling and bandpass filtering to attenuate low-frequency drift and high-frequency noise, complemented by notch filtering for interference suppression. Notably, Avvaru et al. (45) implemented a 0.5 Hz high-pass filter to mitigate low-frequency drift, while McBird et al. (44) employed notch filtering to remove eye blink frequencies. Spatial signal processing involved two critical procedures: bad channel interpolation and reference electrode reconfiguration. A representative approach was demonstrated by Cao et al. (46), who implemented 23 customized bipolar montages to effectively mitigate volume conduction effects in EEG signal analysis. This methodological refinement significantly enhanced spatial resolution while maintaining signal integrity.

#### 4.1.3 Preprocessing of MEG data

One study (47) included in this research utilized MEG data. Similar to EEG, MEG data preprocessing aimed to ensure the accuracy of high-temporal-resolution signals while additionally mitigating environmental magnetic interference and head motion artifacts. For instance, Sami et al. (47) employed temporally extended signal space separation to eliminate environmental artifacts while simultaneously correcting for head displacement. Subsequently, band-pass and notch filtering were applied to remove noise from irrelevant frequency bands (a step analogous to EEG preprocessing). Furthermore, data segmentation, baseline correction, and the removal of physiological artifacts such as eye movements and cardiac activity were crucial for enhancing the reliability of subsequent connectivity analyses.

### 4.2 Extraction of EC features

**Figure 2:**
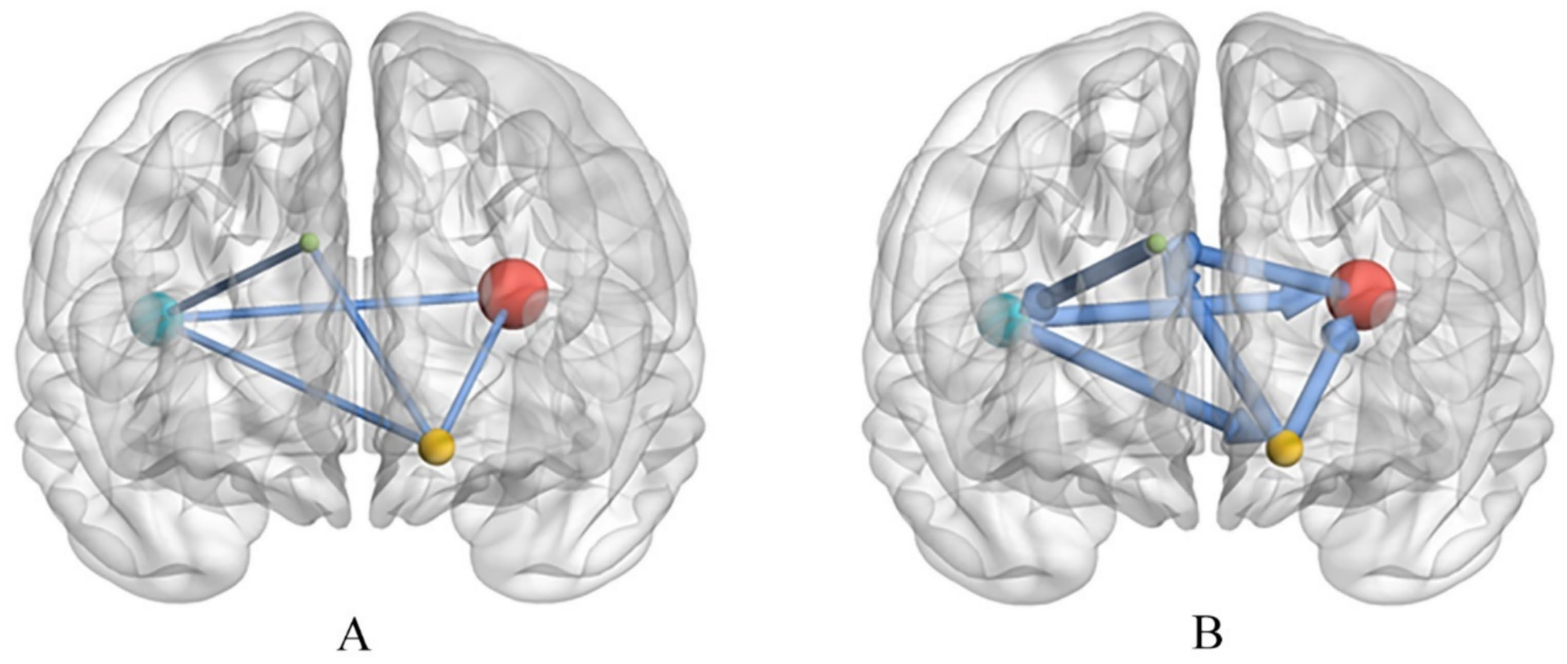
Brain FC network **(A)** and brain EC network **(B)**. **(A)** The functional connectivity network is represented as an undirected graph, where nodes correspond to brain regions, and edges reflect the statistical correlation between regional time series. **(B)** The effective connectivity network is depicted as a directed graph, capturing the direction and strength of information flow between brain regions.

EC reveals directed causal relationships between brain regions by analyzing the temporal dependencies of neural signals. As shown in Figure 2, an effective connectivity network (ECN) comprises nodes representing distinct brain regions interconnected by directed edges. These edges not only delineate structural connections but also quantitatively characterize both the directionality and strength of information flow between neural regions. This deepens our understanding of the brain’s dynamic information processing mechanisms and demonstrates significant potential in neuropsychiatric research. The directional connectivity features inherent in EC exhibit high specificity and sensitivity, making them promising biomarkers for early classification and diagnosis (48). Several studies have employed FC as a comparative model or integrated FC and EC into hybrid models to enhance classification performance (30, 35, 36, 38). As shown in Figure 2, the FC network also consists of two core elements: nodes and edges. The nodes correspond to relevant brain regions, while the edges quantify the strength of FC between brain regions using statistical methods such as Pearson correlation. Unlike directed EC, the undirected edges in FC networks reflect the symmetric nature of connections. FC primarily reflects the correlation of neural activity across regions, typically measured by the synchronization of blood-oxygen-level-dependent signals (49, 50). While FC effectively characterizes functional coordination patterns, it is inherently constrained in uncovering neural regulatory mechanisms due to its inability to infer causality from temporal co-activation alone (51).

**Figure 3:**
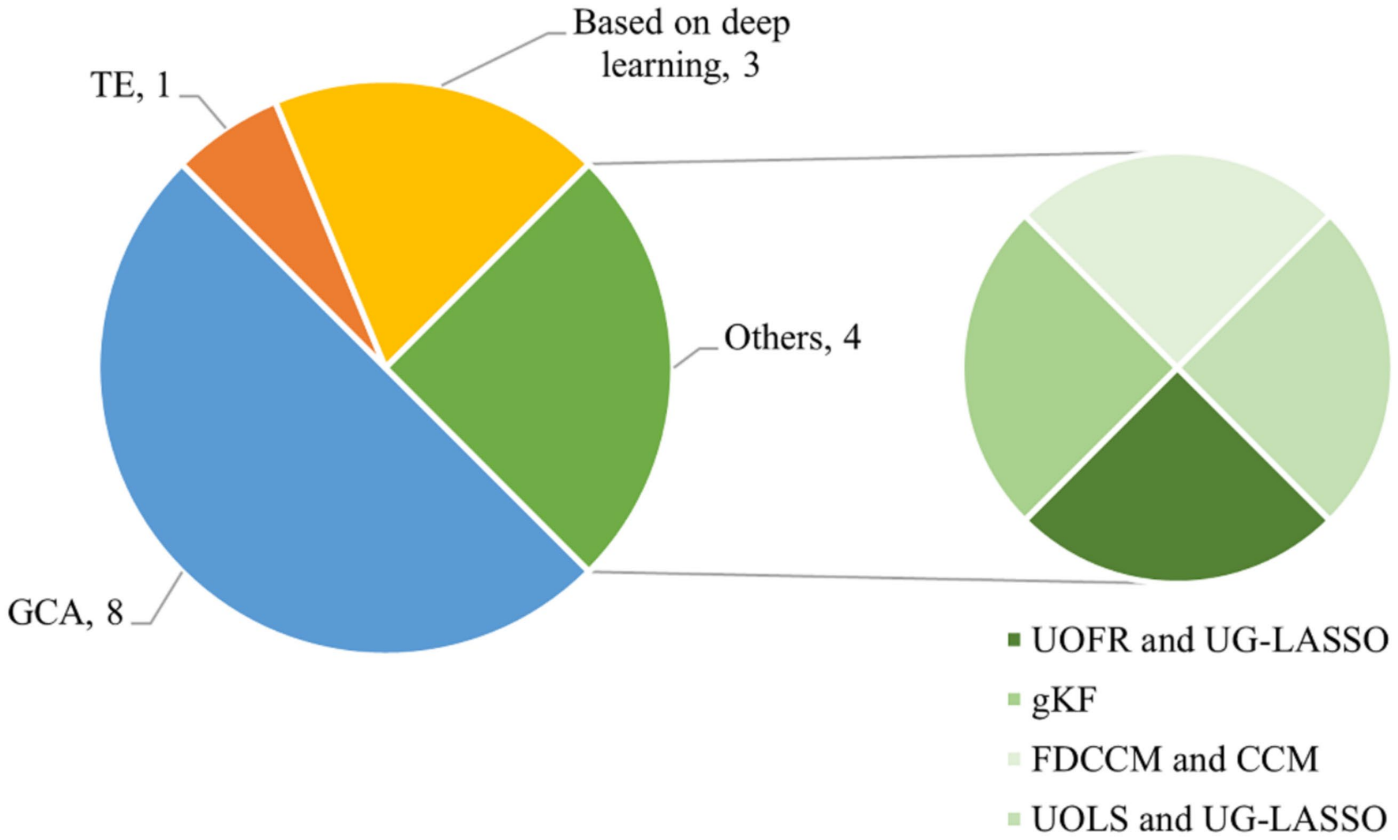
Number of studies for different EC extraction methods. CCM, Convergent Cross-Mapping; FDCCM, Frequency-Domain Convergent Cross-Mapping; GCA, Granger Causality Analysis; gKF, Group Constrained Kalman Filter; TE, Transfer Entropy; UG-LASSO, Ultra-Group LASSO; UOLS, Ultra-Orthogonal Least Squares; UOFR, Ultra-Orthogonal Forward Regression.

Therefore, the accurate quantification of causal interactions between brain regions constitutes the central objective of this study. Figure 3 shows the number of studies for different EC extraction methods. The EC extraction methods used in the 16 selected publications are shown in Tables 1, 2, which can be summarized into Granger causality analysis (GCA), transfer entropy (TE), deep learning–based causal inference, and other emerging techniques for EC estimation.

**Table 1:** Studies of classification model for neurodegenerative diseases based on machine learning and brain effective connectivity.

Study	Date	Feature	Classification model	Validation	Classification situation	The best performing model	
						Model	Accuracy (%)
Li et al. (36)	fMRI	ECs estimated by GCA using MVAR model	SVM	LOOCV	MIC vs. HC		91.89
		FCs estimated by PC					86.49
McBride et al. (44)	EEG	ECs estimated by TE and represented by the mean temporal delays corresponding to peaks in inter-regional	SVM	LOOCV	AD vs. MIC vs. HC		91.7–93.8
Li et al. (37)	fMRI	ECN parameters estimated by UOLS and high-order ECN parameters estimated by UG-LASSO	DCT	10-fold CV	MIC vs. HC		85.5
Khazaee et al. (39)	fMRI	Whole-brain ECs estimated by Multivariate GCA and select directed graph measures as the original feature	NB	10-fold CV	MIC vs. HC		93
Sami et al. (47)	MEG	ECs estimated by graph theory-based measures of local efficiency and used PDC between sensors	SVM	LOOCV	AD vs. HC		85
Guo et al. (33)	fMRI	ECN parameters estimated by BP_KFGC	RBK_SVM	Validation data	AD vs. HC		95.89
Li et al. (38)	fMRI	Whole-brain ECs estimated by GCM	MK-SVM	LOOCV	MIC vs. HC		72.44
		Whole-brain FCs estimated by PC					77.95
		Whole-brain ECs estimated by SR					81.1
		PC and SR					85.83
		PC and GCM					79.53
		SR and GCM					82.68
		MCPC					87.4
Wu et al. (34)	fMRI	dECs estimated by GCA and computed Sample Entropy of the clusters as the classification features	XGBoost, SVM, SVM Cluster, RF	LOOCV	AD vs. HC	SVM	89.8
Hu et al. (35)	fMRI	Whole-brain ECs estimated by RNN-GC	SVM	LOOCV	AD vs. HC		78.72
		Whole-brain FCs estimated by PC					68.09
	sMRI	Cortical measurements estimated by recon-all function in Free Surfer					87.23
	fMRI and sMRI	Whole-brain ECs and cortical measurements					91.49
Zhao et al. (29)	fMRI	dECs estimated by conditional GCA and Fused Lasso	SVM	10-fold CV	AD vs. HC		86.24
		ECs estimated by conditional GCA					80.71
		dECs estimated by conditional GCA and sliding window					82.06
Avvaru and Parhi (45)	EEG	ECN parameters estimated by FDCCM and CCM	NB, SVM, LDA, DT	LOOCV	PD vs. HC	FDCCM-NB	83.9
Wang et al. (32)	fMRI	Whole-brain ECs estimated by GRU_GC	RF, SVM	10-fold CV	EMIC vs. HC	RF	87.88
		Whole-brain ECs estimated by LSTM_GC					62.73
		Whole-brain ECs estimated by MVGC					61.52

AD, Alzheimer’s Disease; BP_KFGC, Back Propagation based Kernel Function Granger causality; CV, Cross-Validation; CCM, Convergent Cross-Mapping; DT, Decision Tree; dEC, Dynamic Effective Connectivity; EC, Effective Connectivity; ECN, Effective Connectivity Network; EEG, Electroencephalogram; EMCI, Early Mild Cognitive Impairment; FC, Functional Connectivity; FDCCM, Frequency-Domain Convergent Cross-Mapping; fMRI, Functional Magnetic Resonance Imaging; GCA, Granger Causality Analysis; GCM, Granger Causality Mapping; GRU-GC, Granger causality with Gate Recurrent Unit; HC, Health Control; LOOCV, Leave-One-Out Cross-Validation; LDA, Linear Discriminant Analysis; LSTM-GC, Long Short-Term Memory-based Granger Causality; MCPC, Multiple Connection Pattern Combination; MCI, Mild Cognitive Impairment; MEG, Magnetoencephalography; MK-SVM, Multi-kernel Support Vector Machine; MVGC, Multivariate Variables Granger Causality; NB, Naïve Bayes; PD, Parkinson’s disease; PC, Pearson’s correlation; PDC, Partial Directed Coherence; RF, Random Forest; RNN-GC, Recurrent Neural Networks Granger Causality; SVM, Support Vector Machine; SVM-Cluster, Support Vector Machine Cluster; sMRI; Structural Magnetic Resonance Imaging; TE, Transfer Entropy; UG-LASSO, Ultra-Group LASSO; UOLS, Ultra-Orthogonal Least Squares; XGBoost, eXtreme Gradient Boosting.

**Table 2:** Studies of diagnosis model for neurodegenerative diseases based on deep learning and brain effective connectivity.

Study	Date	Feature	Classification model	Validation	Classification situation	The best performing model	
						Model	Accuracy (%)
Qiao et al. (30)	fMRI	ECs estimated by GCA	DAG, SVM, LDA, LeNet5, AE	LOOCV	AD vs. HC	DAG	88.24
		ECs estimated by GCA and FCs estimated by 3LHPM-ICA					95.59
Li et al. (40)	fMRI	ECN parameter LE and RCC estimated by UOFR and UG-LASSO	EMLP, MLP, L1-R MLP, L2-R MLP, KNN, RF	LOOCV	MCI vs. HC	EMLP	80.82
		FCs estimated by PC					65.75
Li et al. (41)	fMRI	dECs estimated by gKF and High-order dECs network	VAT-CNN (Extract dEC features) + cwGAT	10-fold CV	EMCIvs. HC		90.9
					EMCIvs. LMCI		89.8
					EMCI vs. LMCI vs. HC		82.7
Cao et al. (46)	EEG	Whole-brain ECs estimated by PDC, GPDC, DTF, ffDTF, and GGC	DSL-GNN, SVM	5-fold CV	AD vs. HC	ffDTF+DSL-GNN	94
					PD vs. HC	ffDTF+DSL-GNN	94.2
					AD vs. PD	ffDTF+DSL-GNN	97.4
					AD vs. PD vs. HC	ffDTF+DSL-GNN	93

AD, Alzheimer’s Disease; AE, Autoencoder; CV, Cross-Validation; cwGAT, Connectivity Weight-Guided Graph Attention Networks; dEC, Dynamic Effective Connectivity; DAG, Directed Acyclic Graph; DSL-GNN Directed Structure Learning Graph Neural Networks; DTF, Directed Transfer Function; EC, Effective Connectivity; ECN, Effective Connectivity Network; EEG, Electroencephalogram; EMCI, Early Mild Cognitive Impairment; EMLP, Elastic Multi-Layer Perceptron; FC, Functional Connectivity; ffDTF, Full Frequency Direct Transfer Function; fMRI, Functional Magnetic Resonance Imaging; GCA, Granger Causality Analysis; GGC, Geweke-Granger Causality; gKF, Group Constrained Kalman Filter; GPDC, Generalized Partial Directed Coherence; HC, Health Control; KNN, K-Nearest Neighbor; LOOCV, Leave-One-Out Cross-Validation; LDA, Linear Discriminant Analysis; LASSO, Least Absolute Shrinkage and Selection Operator; LE, Local Efficiency; LMCI, Late Mild Cognitive Impairment; 3LHPM-ICA, Three-Level Hierarchical Partner Matching Independent Components Analysis; MCI, Mild Cognitive Impairment; MLP, Multi-Layer Perceptron; NB, Naïve Bayes; PD, Parkinson’s disease; PC, Pearson’s correlation; PDC, Partial Directed Coherence; SVM, Support Vector Machine; RCC, Rich Club Coefficients; UOFR, Ultra-Orthogonal Forward Regression; VAT-CNN, Virtual Adversarial Training Convolutional Neural Network.

#### 4.2.1 Granger causality analysis

Among the studies included in this research, eight articles (29, 30, 34, 36, 38, 41, 46, 47) employed GCA to extract EC. In the field of neuroscience, GCA between brain regions is typically modeled using linear autoregressive models. Early studies primarily relied on bivariate autoregressive models to identify causal relationships in the time domain (52, 53), as calculated by Equation (1):


$$X(t)=\sum_{i=1}^{p}a_{i}X(t-i)+\varepsilon_{1}(t)$$

(1) $$X(t)=\sum_{i=1}^{p}a_{i}X(t-i)+\sum_{j=1}^{p}b_{j}Y(t-j)+\varepsilon_{2}(t)$$

Let X(t) denote the observed value of the time series at time t, where $a_{i}$ represents the autoregressive coefficients that quantify the influence of past values X(t−i) on the current value X(t). The terms $\varepsilon_{1}(t)$ and $\varepsilon_{2}(t)$ represent white noise error components, while p denotes the model order. For example, Li et al. (41) employed the dynamicBC (54) toolbox to analyze fMRI data, utilizing its built-in Granger causality (GC) method to derive EC between brain regions. Similarly, Wu et al. (34) estimated whole-brain dynamic EC (dEC) using bivariate GCA within a sliding time window framework. Qiao et al. (30) also applied independent component analysis to extract independent components from fMRI data and constructed an EC matrix based on the GC index, which was used as a feature for classification.

However, this approach struggles to differentiate direct from indirect causal influences, often resulting in false positives (55). To address this limitation, researchers have developed multivariate Granger causality (MVGC) based on multivariate autoregressive (MVAR) models to infer causal relationships among multiple time series. For instance, Zhao et al. (29) applied the Fused Lasso method to identify change points in the connectivity time series, thereby segmenting distinct brain state phases. Within each phase, they employed conditional GC to infer directed dynamic brain networks and construct dEC matrices. Likewise, Li et al. (36) developed a sparse MVAR model based on GC principles to derive directional influence coefficient matrices from fMRI time series as indicators of EC. Moreover, Li et al. (38) utilized MVGC to evaluate EC between brain regions, employing the MVGC toolbox (56) to quantify causal influence strengths among regions of interests. MVGC methods not only mitigate the effects of indirect connections but also reduce external noise, improving the accuracy of EC estimation (56).

Additionally, extending GCA from the time domain to the frequency domain enables the detection of causal relationships across different frequency points or bands, allowing for a more precise characterization of interregional EC (57). Cao et al. (46) introduced a frequency-domain EC analysis method based on the MVAR model, where power spectral density represents network nodes. They integrated five directional connectivity measures to construct brain ECN, achieving superior performance in disease classification. Similarly, Sami et al. (47) utilized MEG data to compute partial directed coherence-based brain networks and assessed EC using graph-theoretical local efficiency measures.

#### 4.2.2 Transfer entropy

One study (44) included in this research employed TE to utilized EC. As a causality metric within the framework of information theory, TE reveals directed information transfer between brain regions by quantifying the extent to which the historical information of variable Y enhances the prediction of the future state of variable X (58). Its core principle lies in evaluating the direction of information flow by computing differences in conditional entropy, mathematically defined by Equation (2):


(2) $$TE_{Y\to X}=\sum p(x_{n},x_{n-d},y_{n-d})\log\frac{p(x_{n}|x_{n-d},y_{n-d})}{p(x_{n}|x_{n-d})}$$

where $x_{n}$ represents the state of variable X at time step n, $x_{n-d}$ and $y_{n-d}$ denote the historical states d steps in the past, $\log p(x_{n}|x_{n-d},y_{n-d})$ is the conditional probability given past information $x_{n-d}$, $y_{n-d}$. McBride et al. (44) utilized TE to evaluate EC among brain regions by extracting time-series signals from multichannel EEG data. They computed TE values to quantify the direction and strength of information flow and employed peak information transmission entropy delays to measure the temporal lag in interregional information transfer. By averaging peak delays across all channel pairs, they derived information transfer characteristics, thereby inferring EC.

#### 4.2.3 Deep learning-based estimation of EC

With advances in deep learning, neural networks are increasingly employed for EC estimation. Among the studies included in this research, three articles (32, 33, 35) integrated neural networks for EC extraction. By integrating GC with neural networks, researchers can better model the brain’s complex causal relationships. Unlike traditional linear GC methods, neural networks effectively capture nonlinear dynamics and handle fMRI data nonstationarity, minimizing regression errors (59). For example, Guo et al. (33) introduced the back propagation-based kernel function GC (BP_KFGC) method, which combines fuzzy inference systems with kernel techniques to enable nonlinear GC analysis through multilayer mapping and hybrid training. Furthermore, neural networks provide greater flexibility in modeling causal relationships over different temporal scales, overcoming the limitations of fixed time lags (60). Hu et al. (35) introduced a recurrent neural network-based GC (RNN-GC) estimation algorithm that utilizes long short-term memory (LSTM) to capture delays in brain signal propagation, constructing dynamic connectivity across whole-brain subnetworks. Wang et al. (32) introduced the gated recurrent unit–GC (GRU_GC) model, which employs GRU to capture dynamic patterns in time series data and constructs an EC matrix for classification purposes.

#### 4.2.4 Other methods for estimating EC features

Recent advances in EC estimation are demonstrated through four (37, 40, 41, 45) innovative methodologies examined in this study. Li et al. (37) pioneered a novel framework integrating low-and high-order ECN through ultra-group lasso (UG-Lasso) and ultra-orthogonal least squares (UOLS) algorithms. The UG-Lasso, applied within sliding windows, robustly estimates stable low-order EC structures, while UOLS provides unbiased quantification of connection strengths. This dual approach enables the construction of high-order ECN whose topological features demonstrate superior performance in MCI classification. Building upon this work, Li et al. (40) initially proposed an ultra-group constrained structure detection algorithm to identify the parsimonious topology of the ECN, employing the ultra-orthogonal forward regression (UOFR) algorithm to construct ECN. Parallel developments in EEG-based EC analysis include Avvaru et al.’s (45) innovative application of convergent cross mapping (CCM) and its frequency-domain extension (FDCCM). This model-free approach uniquely captures nonlinear spectral dynamics in electrophysiological data, revealing hidden causal relationships undetectable by traditional spectral measures. For fMRI data, Li et al. (41) introduced a group-constrained kalman filter (gKF) approach that eliminates spurious connections through sparse regularization while precisely tracking EC temporal evolution.

### 4.3 Representation of EC features

When employed as input features for machine learning and deep learning models, EC can be represented in various forms, including direct variable input, connectivity matrices, and graph-structured data. Among the 16 studies included in this systematic review, approximately 11 adopted the first form—representing EC features as direct variables. These studies (33–35, 37–41, 44, 45, 47) typically extracted key numerical indicators from EC analysis—such as GC values, TE, information flow measures, or graph-theoretical metrics—and used them as input variables for classification models. This method is computationally efficient and easy to implement, making it suitable for traditional machine learning algorithms; however, it may fail to preserve the spatial and topological structure inherent in the original ECN.

In contrast, four studies (29, 30, 32, 36) employed the second approach, representing EC in the form of connectivity matrices. This strategy retains the directional relationships between brain regions, yet remains limited in its ability to capture the dynamic characteristics of brain networks.

Only one study (46) explored the third approach, modeling EC as a directed graph and leveraging a graph neural network (GNN) for classification. This method preserves both the directionality and topological attributes of EC while enabling structural learning across the entire graph, thereby offering superior representational capacity.

## 5 Establishments of classification models

### 5.1 Machine learning models

Machine learning is a branch of artificial intelligence that enables computers to autonomously learn patterns from data (61). Rather than depending on explicitly programmed instructions, it discerns underlying structures in existing datasets to generate predictions or make informed decisions (62). Table 1 summarizes relevant studies on constructing classification models for neurodegenerative diseases using brain EC and machine learning methods.

#### 5.1.1 AD vs. HC

Guo et al. (33) developed a diagnosis model for AD using fMRI-EC and support vector machine (SVM). By leveraging the topological features of ECN, they achieved automatic classification of AD and HC with an accuracy of 95.89%. Wu et al. (34) employed fMRI-EC to classify AD and subsequently developed classification models using various machine learning techniques. Among them, SVM demonstrated the best performance, achieving an accuracy of 89.83% through cross-validation. Hu et al. (35) employed structural MRI (sMRI) and resting-state fMRI (rs-fMRI) to diagnose AD. They extracted fMRI-EC, fMRI-FC, and sMRI-cortical measurements as classification features for a SVM, achieving accuracies of 78.72, 68.09, and 87.23%, respectively. Notably, when fMRI-EC and sMRI-cortical measurements were combined, the classification accuracy improved to 91.49%. Sami et al. (47) developed an AD diagnosis model based on MEG-EC and SVM, achieving a cross-validation accuracy of 85%. Wu et al. (34) utilized fMRI-EC to assess the performance of four classifiers: extreme gradient boosting, SVM clustering, random forest, and SVM. Among these, the SVM classifier achieved the highest classification accuracy of 89.83% when applied to the optimal subset of extracted features. Traditional static functional brain network studies often overlook the rich dynamic information in brain connectivity. Therefore, Zhao et al. (29) developed an AD diagnosis model using fMRI-dEC and applied a SVM for classification, achieving a cross-validation accuracy of 86.24%. This result marks an improvement of 4.18% over conventional dEC models utilizing the sliding window technique and a 5.53% increase compared to models using static EC as features.

#### 5.1.2 MCI vs. HC

Li et al. (36) developed a diagnosis model for MCI based on fMRI-EC, employing SVM as the classifier. The model achieved a cross-validation accuracy of 91.89%, representing a 5.4% improvement over previous methods based on fMRI-FC. Li et al. (37) first built low-order and high-order ECN based on fMRI data, calculated a series of network parameters as features, and used a decision tree to construct a classification prediction model for identifying MCI, with a cross-validation accuracy of 85.5%. Li et al. (37) introduced a novel multi-connectivity pattern combination (MCPC) approach, which integrates three key connectivity features: fMRI-FC, fMRI-EC, and sparse representation. This integrative approach significantly improves the classification accuracy in distinguishing MCI from HC. Experimental results indicate that, when utilizing a multi-kernel SVM classification model, the MCPC method achieves an accuracy of 87.4%, outperforming any individual connectivity model. Wang et al. (32) developed an EMCI diagnosis model based on fMRI-EC and two machine learning methods, with random forest showing the best performance and a test set validation accuracy of 87.88%.

#### 5.1.3 AD vs. MCI vs. HC

Khazaee et al. (39) employed graph metrics as the primary feature set for machine learning algorithms. By utilizing optimal features and a naïve bayes (NB) classifier based on fMRI-EC, they classified AD, MCI, and HC, achieving a cross-validation accuracy of 93.3%. McBride et al. (44) developed a diagnosis model for AD and MCI using EEG-EC and SVM, achieving cross-validation accuracy ranging from 91.7 to 93.8%, depending on the protocol conditions.

#### 5.1.4 PD vs. HC

Avvaru et al. (45) initially constructed a brain ECN based on EEG data, using the network parameters as features to build a PD diagnosis model. The classifiers employed included SVM, linear discriminant analysis, NB, and DT, with NB demonstrating the best performance, achieving a test set validation accuracy of 83.9%.

### 5.2 Deep learning models

Deep learning is a subset of machine learning that involves constructing and training multi-layer neural networks to automatically extract features and patterns from large datasets (63). Unlike traditional machine learning, which relies on manual feature extraction and selection, deep learning reduces the need for human intervention by leveraging the capabilities of neural networks. Table 2 summarizes relevant studies that utilize brain EC and deep learning methods to construct classification models for neurodegenerative diseases.

#### 5.2.1 AD vs. HC

Qiao et al. (30) evaluated various deep learning and machine learning techniques to differentiate AD from HC. The research results indicate that models constructed using directed acyclic graph networks outperform other models. The classification model utilizing fMRI-EC features achieved a cross-validation accuracy of 88.24%, which further improved to 95.59% upon integrating fMRI-FC features.

#### 5.2.2 MCI vs. HC

Li et al. (40) extracted local efficiency and rich club coefficients from brain fMRI-ECN parameters and constructed a diagnosis model for MCI using several multilayer perceptron and machine learning methods. The proposed elastic multilayer perceptron classifier demonstrated superior performance, achieving a cross-validation accuracy of 80.82%. Li et al. (41) extracted global and local features from fMRI-dEC and high-order dECN. They then developed a multi-class prediction model for EMCI, LMCI, and HC classifications using a channel-weighted graph attention network (cwGAT), achieving a cross-validation accuracy of 82.7%.

#### 5.2.3 AD vs. PD vs. HC

Cao et al. (46) first extracted five types of EEG-EC as edges and calculated power spectral density as nodes to form graph data. They then proposed a directed structure learning GNN (DSL-GNN) method to construct a multi-class prediction model to distinguish AD, PD and HC. The results indicated that the model based on full-frequency direct transfer function EC features performed best, achieving a cross-validation accuracy of 93.0%.

## 6 Discussion

This study presents a systematic review of advances in classification models for neurodegenerative diseases, with a particular focus on the integration of EC and machine learning techniques. An analysis of 16 key studies reveals that EC, by elucidating directional and causal information flow between brain regions, offers potential advantages in identifying disease-specific network biomarkers. Currently, an increasing number of classification models based on EC features are being developed using machine learning and deep learning approaches.

The extraction of EC features is a critical component in constructing classification models that integrate EC with machine learning techniques. Current studies predominantly utilize GCA, with bivariate GCA widely adopted for its computational simplicity, despite notable limitations (64). To overcome these constraints, MVGC has gained prominence for its superior ability to model the brain’s nonlinear dynamic properties, thereby improving the accuracy of causal inference and broadening its applicability in classification tasks. Furthermore, advancements such as frequency-domain extensions of GCA and non-parametric approaches like TE have expanded the capacity to quantify nonlinear causal interactions. In recent years, researchers have introduced a range of innovative EC modeling methods, including UG-Lasso/UOLS-based network construction, UOFR constructs ECN by employing a parsimonious topological structure, FDCCM for nonlinear interaction analysis, and gKF for dynamic tracking. Notably, dEC captures temporal fluctuations in brain connectivity and provides novel insights into neural mechanisms. Models based on sliding window analysis, Fused Lasso for detecting state transitions, and deep learning architectures such as RNN-GC and GRU-GC outperform static EC models in classification accuracy, thereby enhancing our understanding of brain dynamics and contributing to more precise classification of neurodegenerative diseases.

In addition to the aforementioned approaches, widely adopted methods for estimating brain EC include structural equation modeling (SEM), dynamic causal modeling (DCM), and Bayesian network modeling (BNM). While these techniques have been extensively applied in the study of other psychiatric disorders, their application in neurodegenerative disease research remains limited (65). Future investigations should consider integrating these methods with machine learning or deep learning frameworks to construct more accurate and robust classification models for neurodegenerative diseases.

Although EC addresses the limitations of FC by elucidating the directional nature of inter-regional neural interactions and enabling causal inference, not all studies consider brain EC as the sole feature for constructing classification models of neurodegenerative diseases (66). Increasingly, research is adopting multimodal data fusion strategies that integrate EC with FC and clinical indicators to enhance model robustness and diagnostic accuracy. For instance, Hu et al. (35) improved the classification accuracy of AD from 78.72 to 91.49% by combining EC features extracted from fMRI with sMRI-based cortical thickness data. Moreover, several studies have demonstrated that combining EC and FC features further improves model performance, largely due to their inherent complementarity (30, 38, 67). Such fusion strategies enable the integration of multidimensional information—including static correlations and causal inferences—thereby overcoming the limitations of single-modality approaches. The MCPC method proposed by Li et al. (38) further supports this advantage, showing a significant boost in classification performance. Multimodal fusion not only capitalizes on the complementary nature of diverse indicators but also facilitates a more accurate characterization of the complex pathological features of neurodegenerative diseases. Additionally, combining traditional EC estimation methods with advanced machine learning or deep learning techniques presents a promising avenue for developing highly accurate and efficient diagnostic models.

In developing classification models for neurodegenerative diseases, both traditional machine learning and deep learning approaches offer unique advantages. Among traditional methods, SVM have consistently demonstrated superior performance due to their effectiveness in handling high-dimensional data (34). In contrast, deep learning techniques exhibit remarkable modeling capabilities through automated feature extraction. Graph-based deep learning approaches, such as GNN, are especially well-suited for inputs in the form of ECN graphs. Notable implementations include the DSL-GNN model incorporating node attributes and directed edge weights, which achieved 93% classification accuracy in distinguishing AD, PD, and HC. Similarly, the cwGAT network integrating dynamic connectivity with higher-order features attained 82.7% accuracy in classifying mild MCI subtypes (EMCI/LMCI/HC). Current research employing deep learning approaches in this domain remains relatively limited, indicating substantial potential for future exploration and advancement.

Current classification models for neurodegenerative diseases based on brain EC show promising results on specific datasets, yet their generalizability across diverse samples and clinical settings remains to be thoroughly validated. For the accuracy of classification models, while a classification accuracy of 90% may be considered an initial benchmark for certain clinical diagnoses, the development of neuroimaging-based classification models demands a more comprehensive evaluation. Beyond achieving high accuracy, it is crucial to assess sensitivity, specificity, and both positive and negative predictive values to ensure the model’s clinical applicability (68). Furthermore, accuracy alone does not substantiate model robustness, as factors such as data dependency, overfitting, and inherent biases may lead to inflated performance estimates. Therefore, a rigorous evaluation framework incorporating multiple performance metrics is essential to provide a more thorough assessment, ultimately enhancing the model’s reliability and clinical credibility (69). On the other hand, the selection of brain atlas significantly affects classifier generalizability, as EC varies with different atlas levels (70). Khazaee et al. (39) compared the performance of the automated anatomical atlas and the 264 putative functional areas atlas for graph node classification, with the latter showing superior classification performance.

Most of the fMRI data employed in this study were derived from the Alzheimer’s Disease Neuroimaging Initiative (ADNI) database (29, 32–35, 38–41). Although ADNI provides a valuable resource for studying disease mechanisms and training predictive models, its reliance on a single data source may constrain the generalizability of the models across diverse populations and clinical settings. Consequently, future research should emphasize the inclusion of multicenter, heterogeneous datasets for external validation and assess model feasibility in real-world clinical environments to enhance generalizability and practical relevance.

Future investigations should consider the following directions: (1) increasing the sample size to enhance model robustness and generalization; (2) advancing brain network modeling and feature extraction methods, with a focus on multi-scale strategies for dEC; and (3) developing integrated models that leverage state-of-the-art machine learning and deep learning techniques to enable more accurate and personalized classification and diagnosis. These efforts will provide a stronger theoretical basis and technical support for the precise identification and intervention of neurodegenerative diseases.

## 7 Conclusion

This study presents a comprehensive review of recent advances in the application of brain EC and machine learning techniques for classifying neurodegenerative diseases. EC, as a key metric for capturing causal interactions between brain regions, has shown promising advantages when utilized as discriminative features in classification model construction. The EC estimation methods adopted in the reviewed studies, along with their practical applications in classification models, are systematically summarized. Future research should prioritize expanding sample sizes to improve model robustness and generalizability, enhancing brain network modeling and feature extraction through multimodal data integration, and developing more efficient ensemble learning frameworks to enable more accurate and individualized diagnostic classification of neurodegenerative disorders.

## Data Availability

The original contributions presented in the study are included in the article/Supplementary material, further inquiries can be directed to the corresponding author.

## Glossary

AD: Alzheimer’s disease
BNM: Bayesian network modeling
BP_KFGC: Back propagation-based kernel function Granger causality
CCM: Convergent cross-mapping
CNN: Convolutional neural network
DCM: Dynamic causal modeling
dEC: Dynamic effective connectivity
DSL-GNN: Directed structure learning graph neural network
EEG: Electroencephalography
EC: Effective connectivity
ECN: Effective connectivity network
EMCI: Early mild cognitive impairment
fMRI: Functional magnetic resonance imaging
FC: Functional connectivity
FDCCM: Frequency-domain convergent cross-mapping
GCA: Granger causality analysis
GC: Granger causality
GCN: Graph convolutional network
gKF: Group-constrained Kalman filter
GRU-GC: Gated recurrent unit Granger causality
HC: Healthy control
HD: Huntington’s disease
LMCI: Late mild cognitive impairment
LSTM: Long short-term memory
MCPC: Multiple connection pattern combination
MEG: Magnetoencephalography
MCI: Mild cognitive impairment
MVAR: Multivariate autoregressive model
MVGC: Multivariate Granger causality
NB: Naïve Bayes
PD: Parkinson’s disease
PRISMA: Preferred reporting items for systematic reviews and meta-analyses
RNN-GC: Recurrent neural network Granger causality
SEM: Structural equation modeling
sMRI: Structural magnetic resonance imaging
SVM: Support vector machine
TE: Transfer entropy
UG-LASSO: Ultra-group least absolute shrinkage and selection operator
UOFR: Ultra-orthogonal forward regression
UOLS: Ultra-orthogonal least squares
